# Bilateral synchronous spermatocytic seminoma: a rare case report

**DOI:** 10.11604/pamj.2014.17.275.4129

**Published:** 2014-04-14

**Authors:** Sankalp Yadav, Nishant Gupta

**Affiliations:** 1 Department of General Surgery, Dr N C Joshi Memorial Hospital, Karol Bagh, New Delhi, India; 2 Department of General Surgery, RML Hospital, President's Estate, New Delhi, India

**Keywords:** Spermatocytic seminoma, testis, testicular seminoma, tumour

## Abstract

Testicular tumors are very common among man under the age of 45 years. The case of bilateral synchronous testicular seminoma is very rare. We present a case of bilateral synchronous testicular seminoma stage-I in a 42-year old Indian male who came to our hospital with chief complaints of dull ache in the abdomen and groin, bilateral scrotal swelling and heaviness, left-sided scrotal swelling since last four years, and right-sided since last two years. He underwent bilateral orchidectomy followed by radiotherapy. In this case we throw light on this rare condition and discuss the management.

## Introduction

Germ cell tumors of testis, in man under the age of 45 years, is the most common malignancy [1]. However, testicular tumors are less common in Asia as compared to the western countries, the incidence is very low as 0.4 per 100,000 population [2]. They are further classified as seminomas and non-seminomas [3]. Seminomas constitute the bulk about 40% of all Germ cell tumors [4, 5]. Seminoma occurring bilaterally is a very rare entity, only seven cases,out which, only two cases from the Indian subcontinent, about this rare condition are published in the literature and are available in PubMed databases [6]. We herein, present a case of very rare bilateral seminoma in a 42 year Indian male with bilaterally enlarged inguinal lymph nodes.

## Patient and observation

Patient 42 year Indian male father of two came to surgery outpatient department of our hospital with chief complaints of dull ache in the abdomen and groin, bilateral scrotal swelling and heaviness, left sided scrotal swelling since last four years and right sided since two years. There was no relevant past, family or personal history that could have been of importance to establish the provisional diagnosis of testicular malignancy. On examination his vitals were stable and there was no cardiovascular, respiratory or neurological disorder. On local examination swelling was present over bilateral hemiscrotum left more than right. On left side swelling was 11X8X6 cm3 (approx.), non-tender, firm to hard in consistency, surface was regular and it was associated with restricted mobility and it was possible to get over the swelling, transillumination was negative. On right side swelling was 8X6X5 cm3 (approx.), non-tender, firm to hard in consistency, surface was regular and it was associated with restricted mobility, it was possible to get over the swelling, transillumination was negative. Testicular sensations on the left side were absent and on the right side were altered (reduced). Also, the inguinal lymph nodes were bilaterally swollen, non-tender and palpable. Patient was admitted to the male surgery ward with a provisional diagnosis of the bilateral testicular malignancy.

Patient was admitted and routine and diagnostic investigations were performed, hemoglobin was 14.6 gm%, Total leukocyte count, differential leukocyte count and platelet count were within their normal range. Serum urea and electrolyte levels were normal. Liver and kidney function tests were within normal limits. Chest radiograph did not reveal any abnormality. Urine routine and microscopic examinations were normal; blood group and cross matching were also done. Special investigations included the assessment of serum tumour markers Beta HCG was elevated 12 mIU/ml, Alpha-fetoprotein and lactate dehydrogenase (LDH) were normal.

**Figure 1:**
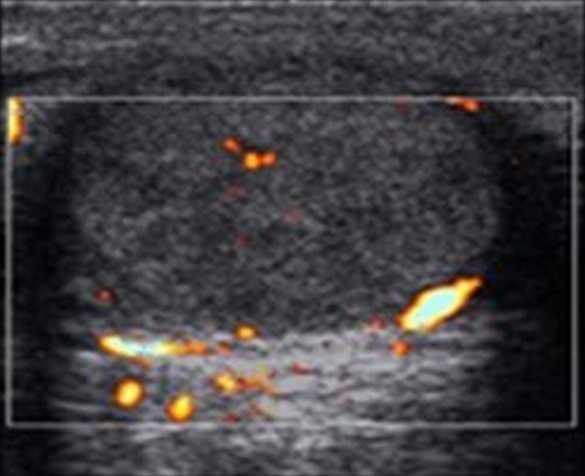
USG image showing enlarged right testis

Ultrasonography of the abdomen and pelvis (Figure 1) revealed the bilateral enlargement of the inguinal lymph nodes with normal echosensitivity and morphology. Testes were multilobulated enlarged and multiple whorled masses were seen scattered all around with peripheral vascularity suggestive of neoplastic nature.

CT scan of the abdomen and pelvis revealed left testis was enlarged measuring about 11X8.8X7.8cm and appears heterogeneously hypodense with a CT attenuation value of 20-30HU on post contrast CT scan there was mild heterogeneous enlargement of the testis with CT attenuation of 30-40HU with few enhancing areas. Right testis was enlarged measuring about 8X6.9X5.5cm and appear heterogeneously hypodense with CT attenuation value of 20-30HU on post contrast CT scan there was mild heterogenous enlargement of the testis with CT attenuation of 30-40HU with few enhancing areas. Both epididymis were not seen separately from the enlarged testis bilaterally. There was moderately non enhancing collection seen within the left extra testicular compartment. There were no internal septations suggestive of left hydrocele. The overlying scrotal wall was thin and stretched.

**Figure 2:**
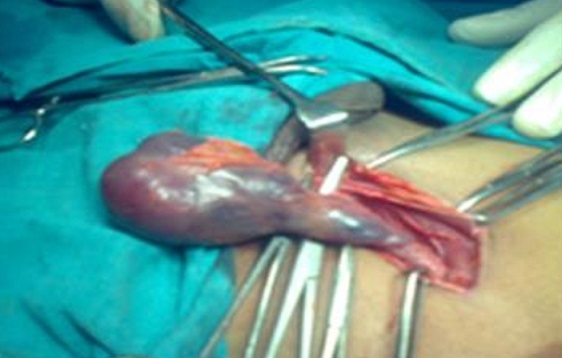
Intraoperative image showing the left testicular mass during left inguinal/radical orchidectomy

Intraoperatively biopsy of the left testis was done from the suspicious area and frozen sections were sent for the histopathological examination. Frozen sections were suggestive of malignancy. Histopathological findings, revealed spermatocytic seminoma stage-IA. The patient underwent Left sided orchidectomy (Figure 2) under general anaesthesia and left testis, spermatic cord and the left inguinal lymph nodes were removed. His postoperative period was uneventful. Sutures removed on the 10th day and the wound was healthy. After 5 days intraoperatively biopsy of the right testis was done and frozen sections were sent for the histopathological examination. Frozen sections were suggestive of malignancy which was later confirmed as spermatocytic seminoma stage-IA, thus confirming bilateral spermatocytic seminoma. Also the histopathological findings were later confirmed by immunohistochemistry. After 15 days Right sided orchidectomy was performed under general anaesthesia and right testis, spermatic cord and the right inguinal lymph nodes were removed. Sutures removed on the 10th day and the wound was healthy.

**Figure 3:**
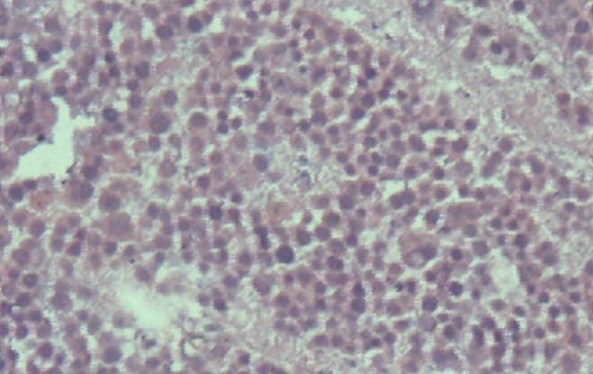
Histopathology picture showing hematoxylin and eosin stained and magnified image with predominant neoplastic cells and inflammatory infiltrates

Histopathological studies revealed that both testes were atrophic and multilobulated. The microscopic evaluation also revealed atrophic seminiferous tubules with reduced spermatogenesis, and dysplastic cells. The seminiferous tubules were surrounded by numerous infiltrates composed of lymphocytes, neutrophils, and macrophages. Neoplastic cells and inflammatory infiltrates were present in both testes (Figure 3).

The patient was given prophylactic radiotherapy for next 15 days and the total dose given was 20Gy to prevent any relapse. He was advised to follow up every 6 months, presently he was asymptomatic, follow-up CECT abdomen and pelvis are normal, tumor markers are well within the limits and there is no evidence of disease.

## Discussion

Germ cell tumors of testis occurring bilaterally is a very rare entity, and its incidence ranges from 1% to 5% as published previously in the large series (Bokemeyeret al, 1993; Dieckmann et al, 1993, 1999, 2002; Heidenreich et al, 1995, 1997, 2000; Gerl et al, 1997; Tekin et al, 2000; Geczi et al, 2001; Ondrus et al, 2001; Che et al, 2002; Ohyama et al, 2002) [7]. Spermtaocytic seminoma stage I is the most common stage of the testicular cancer, as about 80% of all seminomas and 40% of all testicular cancers belong to this group [8]. The important and well established risk factors are cryptorchidism, a previous testicular tumour, family history suggestive of testicular tumors and somatosexual ambiguity syndromes [3]. Besides these, intratubular germ cell neoplasia of the unclassified type is an important precursor. Seminoma seems to be the invasive derivative of intra-tubular germ cell of neoplasia of the unclassified type. Unless complicated by the rare sarcomatous component development, spermocytic seminoma is a nonmetastasizing tumour [3]. The present case is a bilateral synchronous stage-I seminoma. There are very few cases reported in the English literature of this rare tumour. A similar case was reported by Agrawal et al (2010) but this case differs from it, due to the absence of the bilateral cryptorchidism [9]. One more, similar case was reported by Sanjay et al (2012) but this case differs from it in the absence of the bilateral minimal hydrocele and the presence of enlarged bilateral inguinal lymph nodes and raised beta HCG levels, also the patient in this case is a young male of 42 years as against male of 50 years in that case [6].

Management of the bilateral synchronous testicular tumors is a comparatively not easy mainly due to the rarity of the condition and the paucity of the literature. An institutional study carried at Memorial Sloan Kettering Cancer Centre over 50 years, showed 10 patients with synchronous out of 58 reported cases of bilateral testicular germ tumors. The histopathological findings in this study showed seminoma to be the predominant tumor with only 7 cases of nonseminomatous tumor. Treatment as mentioned in this series was mostly based on the histological findings and the stage of the tumor. The patients with both seminomatous and nonseminomatous tumors with stage-I, the method of treatment was retroperitoneal lymph node dissection. Chemotherapy was used in higher stages of tumor. However, stage-I bilateral seminoma were treated with postoperative radiotherapy and in higher stages chemotherapy was the treatment of choice [10].

Over the last 50 years, the main management of testicular stage-I seminoma has been the orchidectomy followed by adjuvant radiotherapy as done in this case. However, many studies mentioned the deadly effects of radiotherapy, which might not even be essential in many cases. The use of surveillance and chemotherapy instead of postoperative radiotherapy, in case of recurrence will be a better option. Radiotherapy has many side effects like nausea, fatigue, oligospermia, increased risk of development of second cancers, peptic ulceration. Besides, there has been lots of questions raised on cardiac toxicity as a side effect of radiations in cancer survivors.

As mentioned by Agrawal et al (2010) due to the presence of lots of studies mentioning about various ways of management only a generalization can be made about the treatment of testicular cancers [9]. Stage of testicular tumor is very important, as seminoma of stage-I should be kept on surveillance and they should be given prophylactic radiotherapy or one to two cycles of adjuvant chemotherapy. The same holds true for the bilateral seminomas but with the only difference that the patient should not be kept on the surveillance, due to the increased load of tumor. Higher stages of tumor should be treated with the chemotherapy. Cases involving small tumors which are limited to the testis, testosterone levels are important in deciding for a testis sparing surgery for the preservation of fertility and to avoid androgen replacement therapy. These cases should be regularly screened for the development of tumor and its metastasis. However the testosterone replacement therapy should be the option for those patients who are not suitable for testis sparing surgery.

Testicular cancer is very common among males but it has very high cure rate in excess of 90%. While most types have a tendency to spread, if not noticed and can spread to the other testicle, and metastasize to the lymph nodes or other body organs, such as the lungs. Early diagnosis and treatment is very important for a favorable outcome. However, in cases where patient failed to come to surgeons, as in this case, there was involvement of both the testes and as the patient had two children i.e. his reproductive life was successful, thus removal of the testes was the best option. It was followed up with a regular radiation therapy and follow up monitoring for any metastasis.

## Conclusion

Testicular malignancies are rare and bilateral synchronous spermatocytic seminoma is the rarest of all the testicular malignancies. It should be considered, especially while evaluating germ cell tumor in both young as well older men. It is distinct in its histologic appearance and can be easily differentiated from other tumors histopathologically. Since, it rarely metastasizes, correct histologic diagnosis and treatment i.e. involving the prophylactic radiotherapy to the lymph nodes involved, followed by orchidectomy for stage I seminoma can have great impact on prognosis. This treatment modality is the most cost-effective and is associated with the lowest risk of tumor recurrence (1% to 3%) [4, 5].
